# Psychometric validation of concerning behavior scale in Iranian children and young people with autism spectrum disorder

**DOI:** 10.3389/fpsyt.2023.1153112

**Published:** 2023-08-03

**Authors:** Kourosh Mohammadi, Abdolvahab Samavi, Fatemeh Zare Mehdiabadi, Seyed AbdolHadi Samavi

**Affiliations:** ^1^Department of Psychology, University of Hormozgan, Bandar Abbas, Hormozgān, Iran; ^2^Department of Educational Sciences, University of Hormozgan, Bandar Abbas, Hormozgān, Iran; ^3^Counseling Department, University of Hormozgan, Bandar Abbas, Hormozgān, Iran; ^4^Department of Educational Psychology, Bandar Lengeh Branch, Isalamic Azad University, Bandar Lengeh, Iran

**Keywords:** assessment of concerning behavior, psychometric validation, people with autism spectrum disorder, validity, reliability

## Abstract

**Introduction:**

Assessment of Concerning Behavior (ACB) was introduced by Tarver et al. (2021) to evaluate mental health and problematic/risky behaviors in children and young people with autism spectrum disorder (ASD).

**Methods:**

This study examined the psychometric validation of the Assessment of Concerning Behavior (ACB) in an Iranian sample of parents of children and young people with ASD. Confirmatory factor analysis was conducted to examine the structure of ACB in a sample of 303 parents.

**Results:**

The data supported the two factor structure, all factor loadings were significant and scale structure was confirmed similar to the original scale. The results supported the two-factor structure for ACB that included internalizing and externalizing problems scales. The two factors of ACB are positively correlated with Aberrant Behavior Checklist scores which showed that the validity of two factors is satisfactory. The reliability of the two subscales was reasonable as well.

**Conclusion:**

The study suggests that the ACB could be an operational tool to assess the mental health and problematic/risky behaviors in Iranian children and young people with ASD.

## Introduction

Autism Spectrum Disorder (ASD) is characterized by impaired communication and social skills, as well as repetitive behaviors and restrictive interests, according to the American Psychiatric Association (1). It is estimated that the global prevalence rate of ASD is approximately 1% (2), while in Iran, the prevalence rate is reported to be 6.26 per 10,000 (3). ASD can have long-term psychosocial consequences and place considerable pressure on individuals with ASD, their families, and caregivers, as well as have economic and social burdens (4).

Research has shown that individuals with ASD often exhibit concerning behaviors that can have negative impacts on their individual and social functioning in the long term (5). Concerning behaviors can also negatively affect the well-being of family and caregivers, as well as society as a whole. Identifying concerning behaviors is crucial as they may indicate underlying co-occurring conditions that require further investigation or may be crucial targets for treatment. Common concerning behaviors observed in individuals with ASD include aggression (6), anxiety (7, 8), hyperactivity (9), compulsive behavior (10), depression (11), and sleep disorders (12).

In addition to the core symptoms of ASD, many children with ASD present with broader behavioral, emotional, and affective problems that require clinical evaluation and intervention. Lindor et al. (13) reported that individuals with ASD often experience clinically significant internalizing and externalizing problems, including depression, anxiety, aggression, somatization, self-injury, inattention, hyperactivity, and impulsivity. Parents and teachers have also reported significant problems with social skills such as cooperation, self-expression, and self-control in children with high-functioning autism or autism spectrum disorder aged 4 to 10 years. These children also display more severe problems such as hyperactivity and internalizing problems compared to their peers (14). According to the Ashburner et al. (15) these problems can include oppositional behavior (39%), lability and regulation (53%), aggressive behavior (14%), and academic problems (54%).

Several studies in the literature have used Aberrant Behavior Checklist (ABC) (16–18) and the Developmental Behavior Checklist (DBC) (19–21) to assess behavioral and emotional problems in individuals with autism. The Aberrant Behavior Checklist (ABC) is a caregiver questionnaire consisting of 58 items designed to evaluate the severity and presence of different problem behaviors commonly noticed in people with developmental and intellectual disabilities (18). Any adult who knows the child well, such as a parent, case worker, teacher, or workshop supervisor, can complete the ABC questionnaire (22).

To the best of our knowledge, there is currently no informant-based questionnaire available in Iran to evaluate concerning behaviors in individuals with ASD. This study aimed to validate the parent/caregiver version of the Assessment of Concerning Behavior (ACB) questionnaire for parents of children and adolescents with autism. Tarver et al. (5) reported that the ACB parent version had a two-factor model (externalizing and internalizing problems) that demonstrated appropriate test–retest reliability, internal consistency and construct validity. Tarver et al. (5) detailed the development and validation of this scale in their paper, which resulted in a 35-item measure with a two-factor model assessing internalizing and externalizing symptoms. The parent-reported version of the ACB demonstrated satisfactory test–retest reliability, internal consistency and concurrent validity.

In our study conducted in Iran, we investigated the Persian version of the Assessment of Concerning Behavior (ACB) questionnaire as a new and favorable tool for clinical and research purposes in autism. The ACB is a mental health assessment scale designed for individuals with ASD and can be used for therapeutic, educational, and research purposes. It has diagnostic value, which can aid psychologists and clinical specialists in diagnosing and designing therapeutic interventions for individuals with ASD. We chose to examine the ACB questionnaire for several reasons. Firstly, it is a relatively new scale, developed in 2022, with good psychometric characteristics in the original sample, despite having fewer questions compared to other accessible scales. Moreover, the ACB questionnaire is capable of measuring a more comprehensive range of high-risk behaviors and coexisting symptoms compared to other questionnaires. Additionally, the ACB questionnaire is easy to use by parents and caregivers.

We validated the parental version of the ACB questionnaire because, in the original study, the parental version had better validity and reliability compared to other versions. Additionally, parents are more accessible than children and adolescents with ASD, and they are the most reliable source for reporting psychological problems and seeking treatment for their children. By examining the reliability and validity of the ACB questionnaire in a sample of Iranian parents of individuals with autism, we established the psychometric properties of the ACB questionnaire in our studied population.

## Methodology

### Procedure

In this study, the psychometric properties of the ACB questionnaire were investigated by translating the original version into Persian using a standardized translation procedure (23). Data were collected through an online survey distributed through social networks and mobile media. The questionnaire was designed and typed on the Porsall website, an Iranian website for online questionnaire design and implementation. The link to access the questionnaire was then provided to participants through social media platforms such as WhatsApp and Telegram.

### ACB scale

In this study, the Assessment of Concerning Behavior (ACB) Scale (5) was used to measure the participants’ opinions regarding the behaviors of children and young people with ASD. The ACB questionnaire was translated into Persian by two bilingual experts (English–Persian) and two external translators, following the guidelines presented by the International Test Commission (24). The translations were reviewed and back-translated into English by a bilingual Doctor of Psychology, who provided terminological adjustments for some terms that were not agreed upon by the previous translators. Although he was not a member of the research team, his input was valuable in ensuring the accuracy of the translated questionnaire.

Participants were asked to match each item on the ACB questionnaire to their child/adolescent’s behavior over the past month, using a five-point Likert scale ranging from 1 (never true) to 5 (always true). The ACB questionnaire includes two dimensions of internalizing and externalizing problems. Externalizing problems (items 1–19) include aggressiveness, control, and impulsivity, while internalizing behaviors (items 20–35) include withdrawal, depression, and anxiety. In Tarver et al.’s study, the Alpha values were 0.86 and 0.88 for the internalizing problems and externalizing problems subscale, respectively (5).

### Participants

The participants in this study were parents of individuals with ASD who were selected through purposive sampling. Informed consent and questionnaires were completed in Persian. A total of 376 parents participated in the study. We excluded cases with inappropriate responses to the questionnaire and distorted questionnaires from the analysis. Inappropriate responses included those with missing or incomplete answers, and distorted questionnaires referred to responses that were highly inconsistent or contradictory. These criteria were applied based on established guidelines for data cleaning and quality control in psychological research. A total of 73 cases were excluded based on these criteria, resulting in a final sample size of *n* = 303. We conducted a non-responder analysis to compare the demographic and baseline characteristics of the excluded participants with those included in the final sample. The results indicated that there were no significant differences between the two groups in terms of age, gender, education level, or any of the main variables of interest, suggesting that the exclusion of these cases did not systematically bias our findings. Also, in the original study, it is noted that the authors have confirmed that “For the majority of items in the parents’ version, most of the responders chose the option ‘not at all’. In the present study, some of the excluded cases were those who had responded ‘not at all’ to all the items in the questionnaire. So, the final sample consisted of 303 parents with an age range of 29–55 years (*M* = 36.48, SD = 4.24). Demographic information, including age, marriage age, socioeconomic status (SES), and the age of the child/adolescent, was collected. Six schools in four cities were purposefully selected to recruit parents of children with ASD, and these schools specialized in providing educational services to children with autism. The age range of the students whose parents participated in the study was between 6 and 15 years, with 41 girls (13%) and 263 boys (87%). Of the students, 223 (73.59%) were classified as requiring support, 73 (24.09%) as requiring substantial support, and 7 (2.31%) as requiring very substantial support. Children and adolescents with ASD who participated in this study were enrolled in schools dedicated to the education of individuals with autism. Before enrolling in these schools, all participants had received a diagnosis of ASD from a psychiatrist and a psychologist. The selected schools provided the parents’ information to the research team, who then contacted the parents and provided information about the purpose of the study. Informed consent was obtained from the participants before they completed the questionnaires, and they were informed that the data would only be used for academic purposes, and their personal information would be kept confidential.

#### Ethics approval

This study was conducted in accordance with the ethical standards of the institutional research committee of the University of Hormozgan, Bandar Abbas, Iran, and with the 1964 Helsinki Declaration and its later amendments. No animal studies were conducted as part of this research. Informed consent was obtained from all participants involved in the study.

### Instruments

#### Aberrant behavior checklist

Aberrant behavior checklist (ABC) was developed by Aman et al. (25) to measure behavior problems across five subscales: lethargy, irritability, stereotypy, inappropriate speech, and hyperactivity/noncompliance. Items were rated on a 4-point Likert scale ranging from 0 (not at all a problem) to 3 (the problem is severe in degree), with higher scores indicating more severe problems. Proper reliability (*α* = 0.86–0.94) and test–retest correlation (approximately 1 month time interval; *r* = 0.96–0.96) were reported across the subscales (17, 25). The items were loaded onto one of five subscales: Irritability, Agitation, and Crying (15 items); Stereotypic Behavior (7 items); Lethargy/Social Withdrawal (16 items); Inappropriate Speech (4 items); and Hyperactivity/Noncompliance (16 items). A total score was then obtained by summing the scores across all subscales. In the present study, the internal consistency of the ABC questionnaire was assessed using Cronbach’s alpha and was found to range between 0.82 and 0.87 across the subscales.

### Data analysis

In this study, descriptive analysis of the items was conducted using SPSS-26. A confirmatory factor analysis (CFA) was performed with the original 2-dimensional (2D) version of the ACB scale using Amos 24.0. The ratio *χ*^2^/*df*, approximation mean square error (RMSEA), and comparative fit index (CFI) were used as fit indices. The model fit was considered good based on the following criteria: a ratio of *χ*^2^ to degrees of freedom greater than 3, a Comparative Fit Index (CFI) of at least 0.90, an SRMR below 0.08, which is a commonly used cut-off for a good fit (26) and a Root Mean Square Error of Approximation (RMSEA) below 0.08, which is a commonly used cut-off for a good fit (27). Convergent validity of the ACB was examined by calculating Pearson’s correlation coefficient with Aberrant Behavior Checklist scores, while reliability was assessed using internal consistency, measured by Cronbach’s alpha coefficient. All analyses were conducted using AMOS-24 and SPSS-26, and the statistical significance level was set at *p* < 0.05 for all analyses.

## Results

### Descriptive results

The means scores of the ACB items and other variables are presented in Table 1.

**Table 1:** Indexes of normality, descriptive statistics, and item analysis of variables.

	Mean	SD	Skewness	Kurtosis
			SE (0.140)	SE (0.279)
1. Spend a lot of the day worried	2.55	0.87	−0.520	−0.642
2. Scared when people that do not know	2.62	0.91	−0.613	−0.450
3. Aches, pains and/or lack energy	2.38	1.02	−0.232	−0.944
4. Dislike him/herself	2.53	0.91	−0.439	−0.702
5. Thoughts and beliefs which are not real	2.44	0.95	−0.335	−0.806
6. Hard to be happy with self or other people	2.43	0.88	−0.315	−0.637
7. Ritual that must do to stop feeling upset	2.52	0.93	−0.504	−0.566
8. Worry about getting fat	2.48	0.93	−0.306	−0.824
9. Think and behave in set way	2.43	0.96	−0.269	−0.819
10. Dislike being separated from certain people	2.57	0.87	−0.508	−0.501
11. See or hear things that others cannot	2.59	0.98	−0.494	−0.740
12. Nightmares	2.49	1.01	−0.390	−0.824
13. Scared of animals or situations	2.51	0.96	−0.356	−0.824
14. Senses seem to bother	2.45	0.96	−0.274	−0.820
15. Stopped enjoying things or lost interest	2.68	1.01	−0.629	−0.767
16. Hard to wake up sleepy during the day	2.59	1.01	−0.485	−0.942
17. Part of body hurts or itches	2.32	0.94	−0.219	−0.810
18. Very interested and think about a lot of time	2.58	1.04	−0.509	−0.881
19. People force to do things that does not want	2.58	1.02	−0.458	−0.869
20. Hit or hurt people	2.46	0.95	−0.430	−0.665
21. Damage items	2.27	0.84	−0.059	−0.853
22. Refuse to follow rules	2.41	0.92	−0.302	−0.617
23. Does things without thinking	2.43	0.94	−0.286	−0.687
24. Enjoy hurting people or animals	2.35	0.93	−0.273	−0.660
25. Shout at or threaten	2.60	1.01	−0.445	−0.878
26. Too much energy	2.82	0.92	−0.802	−0.364
27. Mood changes very quickly	2.76	1.02	−0.738	−0.405
28. Control people	2.75	0.94	−0.745	−0.277
29. Does not care to upset	2.78	0.99	−0.745	−0.454
30. Short attention span	2.72	0.94	−0.716	−0.359
31. Hurt or injure	2.84	0.98	−0.841	−0.313
32. Eat too much	2.83	0.96	−0.796	−0.365
33. Do not acceptable things on the internet	2.63	1.02	−0.541	−0.691
34. Movements speeded up or slowed down	2.73	0.97	−0.686	−0.506
35. Sexual behaviors bother others	2.63	0.96	−0.548	−0.712
Internalizing	47.82	4.61	−0.176	−0.871
Externalizing	42.08	3.98	−0.611	−0.306
ABC total score	104.63	6.65	−1.040	0.434

### Results of confirmatory factor analysis

In this study, a confirmatory factor analysis (CFA) with maximum likelihood estimation was conducted to examine the factor structure of the ACB questionnaire. In confirmatory factor analysis (CFA), maximum likelihood (ML) is commonly used and assumes that the observed indicators follow a continuous and multivariate normal distribution. We tested three models: one factor, two factors, internalizing and externalizing models. The results indicated that all factor loadings were significant and that the scale structure was consistent with the original scale, with two dimensions of internalizing and externalizing problems subscales. The model fit indices, presented in Table 2. According to fit indices in Table 2, the two-factor model has a better fit compared to other models. Therefore, the two-factor model was proposed as the final model. Table 3 provides the factor loadings of the scale items, showing that all factor loadings, were significant and higher than 0.30.

**Table 2:** Model fit indexes of one factor, two factors, internalizing and externalizing models.

Model	Index	*χ* ^2^	*df*	*χ*^2^/*df*	RMSEA (CI 95%)	SRMR	CFI
One factor	Values	4008.205	546	4.74	0.143 [0.139; 0.147]	0.174	0.712
Two factors	Values	1565.000	558	2.80	0.077 [0.073; 0.082]	0.066	0.916
Internalizing	Values	380.160	104	3.65	0.094 [0.084; 0.104]	0.044	0.940
Externalizing	Values	714.044	152	4.69	0.111 [0.103; 0.119]	0.046	0.920

**Table 3:** Factor loadings of the scale items.

Factors/Items		Loadings	*p*
Internalizing factor	1. Spend a lot of the day worried	0.896	0.001
	2. Scared when people that do not know	0.919	0.001
	3. Aches, pains and/or lack energy	0.850	0.001
	4. Dislike him/herself	0.865	0.001
	5. Thoughts and beliefs which are not real	0.892	0.001
	6. Hard to be happy with self or other people	0.889	0.001
	7. Ritual that must do to stop feeling upset	0.883	0.001
	8. Worry about getting fat	0.842	0.001
	9. Think and behave in set way	0.858	0.001
	10. Dislike being separated from certain people	0.835	0.001
	11. See or hear things that others cannot	0.838	0.001
	12. Nightmares	0.821	0.001
	13. Scared of animals or situations	0.839	0.001
	14. Senses seem to bother	0.825	0.001
	15. Stopped enjoying things or lost interest	0.854	0.001
	16. Hard to wake up sleepy during the day	0.811	0.001
	17. Part of body hurts or itches	0.842	0.001
	18. Very interested and think about a lot of time	0.829	0.001
	19. People force to do things that does not want	0.852	0.001
Externalizing factor	20. Hit or hurt people	0.844	0.001
	21. Damage items	0.766	0.001
	22. Refuse to follow rules	0.826	0.001
	23. Does things without thinking	0.800	0.001
	24. Enjoy hurting people or animals	0.774	0.001
	25. Shout at or threaten	0.776	0.001
	26. Too much energy	0.822	0.001
	27. Mood changes very quickly	0.839	0.001
	28. Control people	0.827	0.001
	29. Does not care to upset	0.839	0.001
	30. Short attention span	0.824	0.001
	31. Hurt or injure	0.865	0.001
	32. Eat too much	0.845	0.001
	33. Do not acceptable things on the internet	0.801	0.001
	34. Movements speeded up or slowed down	0.839	0.001
	35. Sexual behaviors bother others	0.821	0.001

Table 2 presents the results of the confirmatory factor analysis for the measurement models of the Assessment of Concerning Behavior (ACB) questionnaire. The measurement models were checked first to ensure that the items loaded well on their respective factors before proceeding to the testing of the structural model. Model 1 represents the single-factor model, Model 2 represents the two-factor model, and Model 3 and 4 represents the internalizing and externalizingmodels.

### Convergent validity and reliability

To assess the reliability of the ACB questionnaire and its two dimensions, Cronbach’s alpha coefficients were calculated. Convergent validity was examined by investigating the correlation between ACB scores and ABC scores. The results showed a significant correlation between the internalizing and externalizing subscale subscales and ABC scores. The correlation coefficients and Cronbach’s alpha values are presented in Table 4. Convergent validity and reliability were assessed to determine the extent to which the Assessment of Concerning Behavior (ACB) questionnaire measures the same construct across different measurement tools and methods. The results showed that the ACB questionnaire has good convergent validity and reliability, as indicated by the reasonable correlations with Aberrant Behavior Checklist score, and the high internal consistency of the ACB subscales.

**Table 4:** Assessment of concerning behavior (ACB) convergent validity and reliability (Cronbach’s alpha coefficient).

	*α*	1	2	3
1. Internalizing subscale	0.88	1		
2. Externalizing subscale	0.94	0.643^**^	0.1	
3. Aberrant Behavior Checklist	0.85	0.513^**^	0.796^**^	1

** Correlation is significant at the 0.01 level (2-tailed).

## Discussion

The objective of the current study was to evaluate the psychometric properties of the ACB questionnaire in a sample of Iranian parents of individuals with ASD, and to establish an appropriate solution for this population. Additionally, we examined the association between ACB scores and a source of convergent validity (ABC scores). The research findings confirmed the validity and reliability of the ACB questionnaire. The results of the confirmatory factor analysis (CFA) supported the two-factor structure of the ACB, consisting of internalizing and externalizing problems subscales. This structure aligns with the current conceptualization of concerning behaviors, which includes both intrapersonal and interpersonal aspects, such as worrying (intrapersonal) and hitting or hurting others (interpersonal) (28). The challenges that individuals with ASD face in their relationships with others are marked by deficiencies in social interaction, communication, and imitation, as well as physical or verbal aggression, conduct issues, and hyperactive behaviors that can disrupt activities (29). These challenges may involve behaviors such as breaking rules, disturbing others, and interfering with activities. According to Shea et al. (29), socialization scores are significantly linked to externalizing behaviors in people with ASD. Volker et al. (30) found that individuals with ASD show more externalizing behaviors compared to peers across all cognitive levels. On the other hand, internalizing problems such as depression, anxiety, and withdrawal have a greater impact on an individual’s internal psychological state rather than the external environment (31). Our research, similar to the original scale developed by Tarver et al. (5), indicates that the ACB measures both internalizing and externalizing issues.

Moreover, the reliability of the internalizing and externalizing subscales was assessed through the calculation of Cronbach’s alpha coefficient, which indicated that these subscales had appropriate alpha coefficients. Additionally, the correlation of the internalizing and externalizing subscales with ABC scores provided evidence of convergent validity. The results showed a significant relationship between the ABC scores and the internalizing and externalizing problems subscales of the ACB. Overall, the analyses in our study confirmed the reliability and validity of the internalizing and externalizing subscales, which is consistent with previous research (5, 32).

Despite the ACB being a valid tool for assessing the psychological problems of children and adolescents with ASD, there are some limitations to its use, particularly in versions designed for teachers and individuals with autism. The reliability and validity of the autistic children and young people version of the scale have been reported as less satisfactory, and low completion rates on the child and teacher versions are significant limitations. Autistic individuals may have difficulty understanding the scale items due to cognitive and communication limitations, making it difficult to obtain accurate responses. Conversely, parents and caregivers of individuals with ASD spend the most time with them and are a credible source of information for evaluating their mental health. The Persian version of the ACB has the potential to provide a comprehensive assessment of concerning behaviors in individuals with ASD, which can assist psychologists and clinical professionals in developing treatment and educational interventions to improve their mental health.

The developers of the ACB conducted analyses to explore the relationships between scores on the ACB scales and the sex and age of individuals with ASD and found no differences in parent-reported externalizing behavior on the ACB based on sex. Similar to the original developers of the scale, we believe that the ACB is suitable for use across the age-span, particularly for individuals between 7 to 29 years old. However, it is recommended that future studies investigate the appropriateness of the ACB for different age and IQ groups.

### Implications

The findings of our study have significant implications for scholars and practitioners as it provides a simple and psychometrically comprehensive tool for measuring concerning behaviors in children and young people with ASD. Additionally, this scale can be used to examine the relationship between concerning behaviors and other psychological and emotional constructs in individuals with ASD. Future studies could investigate the relationship between concerning behaviors and parenting stress, self-regulation problems, and academic outcomes. Furthermore, the ACB can be used to assess the effectiveness of interventions aimed at improving the mental health of individuals with ASD. To increase the generalizability of our findings, future studies could examine the validity of the ACB using exploratory factor analysis (EFA).

### Limitations

Although significant information is provided in this study, however, there are some limitations to our study. Firstly, our sample comprised Iranian parents of individuals with ASD, and caution should be exercised when generalizing the findings to other populations. Secondly, data collection was conducted online, and participants answered the questionnaire voluntarily, which may introduce bias. Finally, our study only examined the ACB’s convergent validity with ABC scores, and future studies could investigate its divergent validity by studying its correlation with variables such as social skills, well-being, and happiness.

## Data availability statement

The raw data supporting the conclusions of this article will be made available by the authors, without undue reservation.

## Ethics statement

The studies involving human participants were reviewed and approved by Hormozgan University. The patients/participants provided their written informed consent to participate in this study.

## Author contributions

KM, AS, FZ, and SAS: conception and design. SAS and FZ: data collection, KM, AS, FZ, and SAS drafting manuscript, and agreeing final submission. SAS: data analysis. KM and AS: interpretation of results. All authors contributed to the article and approved the submitted version.

## Conflict of interest

The authors declare that the research was conducted in the absence of any commercial or financial relationships that could be construed as a potential conflict of interest.

The reviewer KH declared a past co-authorship with the author AS to the handling editor.

## Publisher’s note

All claims expressed in this article are solely those of the authors and do not necessarily represent those of their affiliated organizations, or those of the publisher, the editors and the reviewers. Any product that may be evaluated in this article, or claim that may be made by its manufacturer, is not guaranteed or endorsed by the publisher.
